# Acclimation in intertidal animals reduces potential pathogen load and increases survival following a heatwave

**DOI:** 10.1016/j.isci.2023.106813

**Published:** 2023-05-04

**Authors:** Elliot Scanes, Nachshon Siboni, Brendon Rees, Justin R. Seymour

**Affiliations:** 1 Climate Change Cluster, University of Technology Sydney, Ultimo, NSW 2007, Australia; 2 Sydney Institute of Marine Science, Mosman, NSW 2088, Australia

**Keywords:** Marine organism, Global change, Biological sciences

## Abstract

Intertidal animals can experience intense heat during a heatwave, leading to mortality. The causes of death for intertidal animals following heatwaves have often been attributed to a breakdown in physiological processes. This, however, contrasts with research in other animals where heatwave mortality is attributed to existing or opportunistic diseases. We acclimated intertidal oysters to four treatment levels, including an antibiotic treatment, and then exposed all treatments to a 50°C heatwave for 2 h, replicating what can be experienced on Australian shorelines. We found that both acclimation and antibiotics increased survival and reduced the presence of potential pathogens. Non-acclimated oysters had a significant shift in their microbiome, with increasing abundances of bacteria from the Vibrio genera, including known potential pathogens*.* Our results demonstrate that bacterial infection plays a pivotal role in post-heatwave mortality. We anticipate these findings to inform the management of aquaculture and intertidal habitats as climate change intensifies.

## Highlights

• Acclimation reduced oyster death following an atmospheric heatwave
• Non-acclimated oysters had more bacteria from the genus Vibrio in their tissues
• Acclimated oysters had greater numbers of circulating hemocytes (white blood cells)
• Bacteria play a major role in post-heatwave mortality

## Graphical abstract

**Figure:**
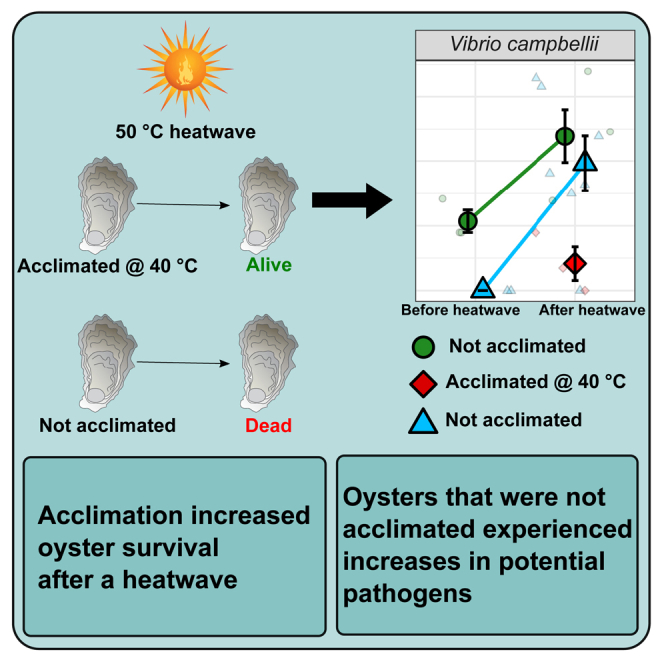

## Introduction

Heatwaves are periods of anomalous warming that occur across both terrestrial and marine habitats, often with fatal consequences.^1^ In intertidal marine habitats, heatwaves can manifest as both marine heatwaves (elevated water temperature) or atmospheric heatwaves (elevated air temperature). Atmospheric heatwaves can raise the temperature of the substratum to 60°C in some cases,^2^ resulting in widespread mortality of intertidal organisms.^3^ As atmospheric heatwaves cause extreme heating, intertidal organisms must endure these temperatures while out of the seawater (i.e. *emersed),* which is their most vulnerable time.^4^^,^^5^ Heatwaves are increasing in frequency and severity as a result of climate change,^6^ but there is very little knowledge on how intertidal marine species respond, adapt, and overcome such devastating events.

Intertidal habitats are globally important hotspots of marine biodiversity, harboring a diversity of functional groups and specially adapted organisms in a relatively small area.^7^ The diversity of intertidal habitats stems from their heterogeneity, whereby heat extremes can vary due to height on the shore, orientation, or the presence of overhangs and crevices.^8^ This heterogeneous habitat has led sessile intertidal organisms to require high degrees of phenotypic plasticity.^9^ Sessile intertidal organisms like oysters can settle and metamorphose anywhere in the intertidal zone, meaning that larval oysters must be genetically equipped to deal with a diversity of possible future habitats.^4^^,^^10^ Therefore, a large proportion of their genome is devoted to phenotypic plasticity and coping with environmental stress.^9^ Plasticity has given oysters the ability to acclimate physiological processes in accordance with their environment.^4^^,^^11^ Acclimation, however, comes at a cost. Acclimation to thermal stress requires the production of molecular defenses, including heat shock proteins (HSPs) that divert energy away from other physiological processes^11^ and may result in ecological trade-offs such as slower growth rates.^12^

Acclimation to heatwaves that increases survival among oysters has largely been explained by acute thermal tolerance and physiological mechanisms to guard against thermal damage to cells and cellular processes.^13^^,^^14^^,^^15^^,^^16^ However, there is growing evidence that disease may play a larger role than previously considered. For example, in humans, most hospital admissions and deaths during an atmospheric heatwave are due to existing chronic disease that is exacerbated by heatwave conditions.^17^ In the marine environment, marine heatwaves have been implicated in outbreaks of sea-star wasting disease, which has resulted in the collapse of populations of this keystone predator.^18^ Despite this obvious link, there is limited evidence for the interaction between heatwaves and disease in marine systems, especially for intertidal organisms. In one of the few studies to investigate the role of disease in intertidal oyster mortality, it was found that mortality could be avoided when bacteria were removed by adding antibiotics during a marine heatwave.^19^

The role of microorganisms including bacteria, viruses, and fungi is increasingly recognized in shaping the health and survival of marine invertebrates.^20^ Marine invertebrates, including oysters, harbor a diverse community of microorganisms in their microbiome with both pathogenic and beneficial bacteria present.^21^^,^^22^ However, changes in the external environment can shift the microbiome, leading to increases in the abundance of potential pathogens.^19^^,^^22^ Bacteria from the Vibrionaceae genus (Vibrio) are ubiquitous marine bacteria that can often form pathogenic associations with oysters and other marine invertebrates. Vibrio species can act as primary pathogens, leading to disease in healthy invertebrates, or opportunistic pathogens, causing disease when invertebrates’ protective barriers are weakened or their immune system is suppressed.^23^^,^^24^ Infection by pathogenic Vibrios is, therefore, dependent on host and environmental factors.^25^ Environmental disturbances such as warming can trigger infection by pathogenic Vibrios by reducing the immune defenses of the host,^25^^,^^26^ and favor the growth of Vibrios, which are strongly thermally dependent.^23^ Vibrio species from the *splendious* and *harveyi* clades have been shown to possess particular pathogenicity to oysters and other marine invertebrates, and are considered responsible for significant mortality events in aquaculture.^27^^,^^28^

Vibrios and other bacteria can play a significant role in shaping the health and survival of oysters and intertidal communities; however, it remains unknown how pathogenic bacteria may influence oyster survival following an atmospheric heatwave, and how these interactions can be mediated by acclimation. Oysters play an important role in forming intertidal habitat on shorelines around the globe. Intertidal shorelines in eastern Australia are often dominated by the habitat forming Sydney rock oyster *Saccostrea glomerata* that is the basis of the Australian oyster aquaculture industry with a total value of $300 million to the state of NSW when secondary services are considered.^29^ *S. glomerata* can live across a range of tidal elevations where they may be forced to acclimate to different thermal regimes.^30^ There is evidence that *S. glomerata* is highly vulnerable to atmospheric heatwaves with an apparent genetic basis to their vulnerability.^31^ *S. glomerata* has also displayed a strong capacity for phenotypic plasticity, whereby oysters are able to adjust metabolic and clearance rates with changes in temperature.^4^^,^^15^^,^^32^ This study sought to determine the role played by the immune system and potentially pathogenic bacteria in thermal acclimation and mortality following an atmospheric heatwave. We hypothesized that survival of *S. glomerata* following exposure to a heatwave would be improved following prior acclimation, and survival would be influenced by the bacterial communities present in oysters. We tested this hypothesis by exposing oysters to different acclimation regimes and then a 50°C atmospheric heatwave, which is characteristic of intense thermal conditions often experienced during the Australian summer.

## Results

### Heatwaves reduce survival

**Figure 1:**
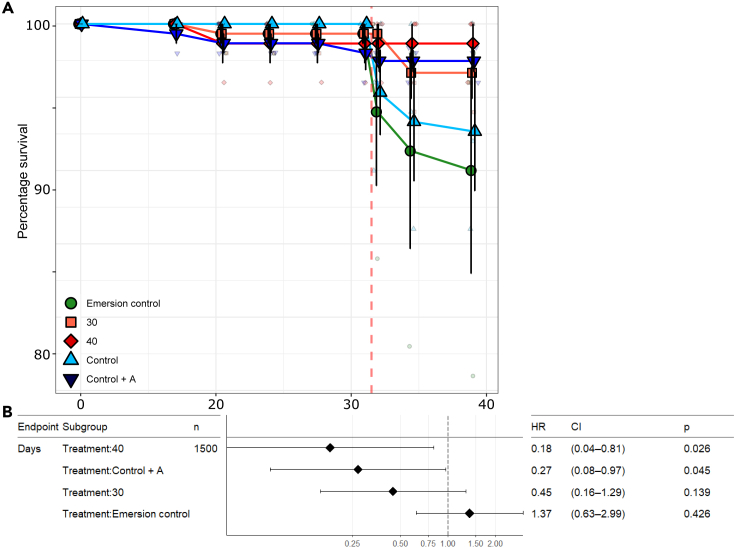
Survival of oysters (A) Mean ± SE cumulative percentage survival of *S. glomerata* over the experimental period. Red dashed line indicates the 50°C heatwave. Colors and shapes indicate the acclimation treatments of oysters. N = 3. Small points indicate raw data points. (B) Forest plot of Cox proportional survival results. Points indicate the Hazard Ratio (HR) compared to the Control treatment (HR = 1). HR scores less than 1 indicate a reduced risk of death compared to the Control treatment. Error bars indicate 95% confidence intervals.

Exposure to a 50°C heatwave caused oyster mortality in the Control and Emersion control treatments (Figure 1). The Cox proportional survival model indicated that there was a significant effect of the treatments on oyster survival throughout the experiment. Oysters in the 40°C and Control + antibiotic treatments displayed significantly greater probabilities of survival compared to the Control treatment. Oyster survival in the Emersion control and 30°C treatments were not significantly different from the Control (Figure 1B).

### Oyster growth is affected by acclimation dependant on treatment levels

Heatwave acclimation treatments significantly affected the rate of growth of *S. glomerata*. Overall growth was greatest in the 40°C treatment which was significantly greater than the 30°C treatment but not the Control or Emersion control (AVOVA F = 2.6_3,464,_ p < 0.05). Over the 5 weeks prior to the heatwave, oysters averaged an increase of 19.6 ± 5.5 mm^2^ (mean ± SE), followed by 16.1 ± 4.2 mm^2^ in the 30°C treatment, 16 ± 4.9 mm^2^ in the control, and 14 ± 3.9 mm^2^ in the 20°C treatment. These effects were not consistent among the weeks, with the rate of growth in the control treatment remaining steady during the experiment, while growth rates of oysters in the acclimation treatments varied (ANOVA; Time × Treatment; F = 10_9,464_, p < 0.001). Pairwise tests at each measurement time point showed that after two weeks (one week of acclimation treatments) growth was significantly greatest for oysters in the 30°C treatment. In the following week (week 3; 2 weeks of acclimation treatments), the 30°C treatment had the lowest rates of growth, followed by the Emersion control treatment, while the 40°C treatment and Control were not different. By week 4 (3 weeks of acclimation treatments), the rate of growth in all acclimation treatments was significantly greater than the Control treatment. By 5 weeks (4 weeks of acclimation treatments), however, there were no significant differences in growth rates among the treatments or Controls (Figure S1).

### Total hemocyte and granulocyte counts increase with acclimation

**Figure 2:**
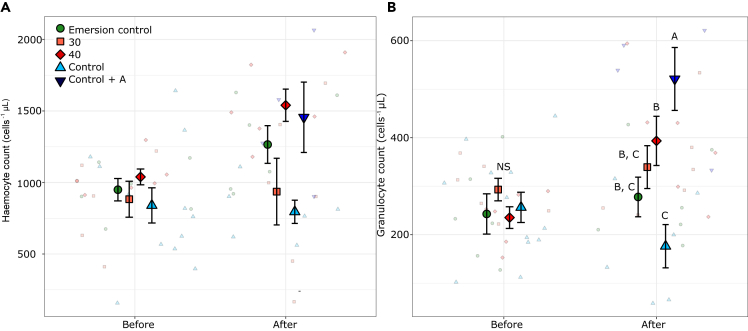
Oyster hemocyte counts (A) Mean ± SE hemocyte count (cells^−1^ μL) and (B) Mean ± SE granulocyte count (cells^−1^ μL) counted in *S. glomerata* hemolymph using flow cytometry before and after the 50°C atmospheric heatwave. Colors and shapes indicate acclimation treatments. Letters above points indicate significant differences among treatments as determined by post hoc tests (p < 0.05) before and after the heatwave, NS = Not Significant. N = 6. Small points indicate raw data points.

The total hemocyte count (THC) was significantly different among treatments (Figure 2A; ANOVA; F_4,49_ = 5, p < 0.05). Oysters from the 40°C and Control + antibiotic treatments had a significantly greater THC than the Control oysters (Figure 2A). There was no interaction effect between the heatwave and acclimation treatments on THC.

Acclimation treatments significantly affected the total granulocyte count of *S. glomerata* individuals (ANOVA; Treatment × Heatwave interaction; F_4,49_ = 3.1, p < 0.05), with this pattern exacerbated by the 50°C heatwave treatment (Figure 2B). Before the heatwave, there were no significant differences in granulocytes among the treatments (Figure 2B). After the heatwave, granulocytes were elevated in the 40°C and Control + antibiotics treatments, both of which were significantly greater than the Control treatment (Figure 2B).

### Total Vibrio abundance increases following heatwaves

**Figure 3:**
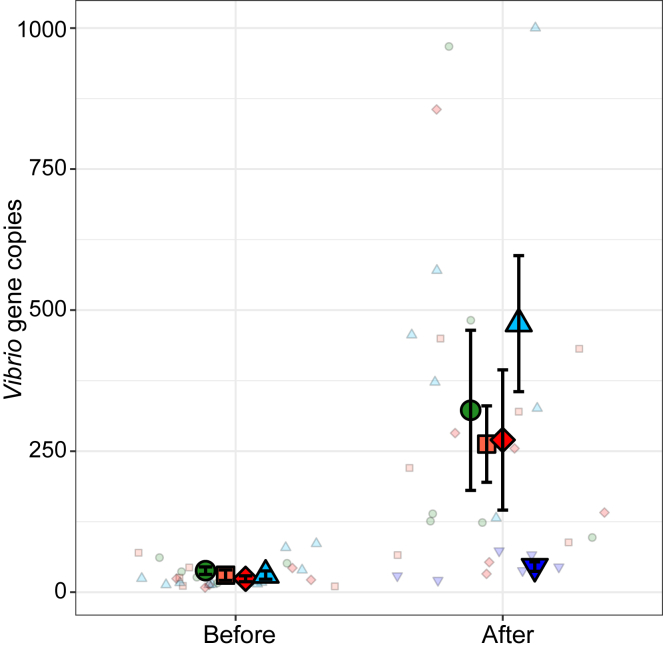
Vibrio bacterial abundance Mean ± SE Vibrio gene copies in *S. glomerata* gill tissue determined by qPCR before and after the 50°C atmospheric heatwave. Colors and shapes indicate the acclimation treatments of oysters. N = 6. Small points indicate raw data points.

The 50°C heatwave triggered a significant increase in the number of Vibrio bacteria (Chi^2^ ANOVA; LR = 152, p < 0.01). This increase was greatest in the control treatment, and lowest in the Control + antibiotic treatment (Figure 3).

### Bacterial community composition is altered by heatwaves and acclimation

**Figure 4:**
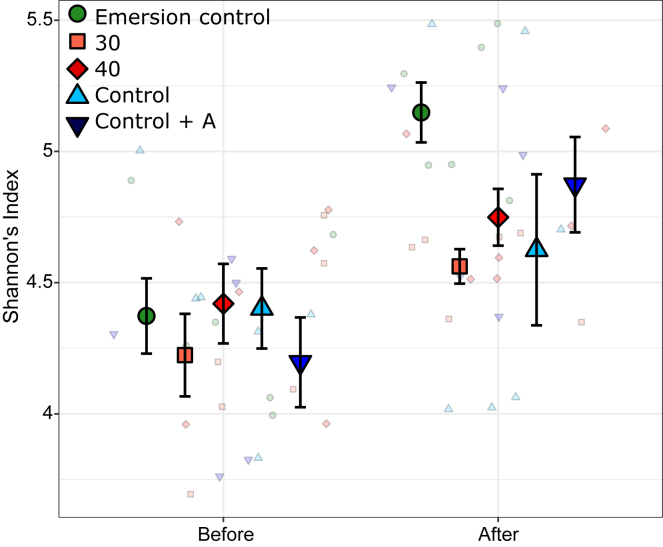
Bacterial diversity Mean ± SE Shannon’s index of bacterial diversity before and after the 50°C atmospheric heatwave in oyster gill tissue. Colors and shapes indicate the acclimation treatments of oysters. N = 6. Small points indicate raw data points.

Oyster bacterial communities were largely dominated by spirochetes. The addition of antibiotics drastically altered this community with spirochetes replaced by bacteria from the family Endozoicomonadaceae (Figure S3). Bacterial diversity (Shannon’s) and Evenness (Pielou’s) were not significantly different among acclimation treatments; however, the 50°C heatwave caused a significant increase in bacterial diversity (ANOVA F_4,48_ = 1.4, p < 0.01) and evenness (ANOVA F_4,48_ = 7.08, p < 0.01) across all acclimation treatments (Figure 4).

**Figure 5:**
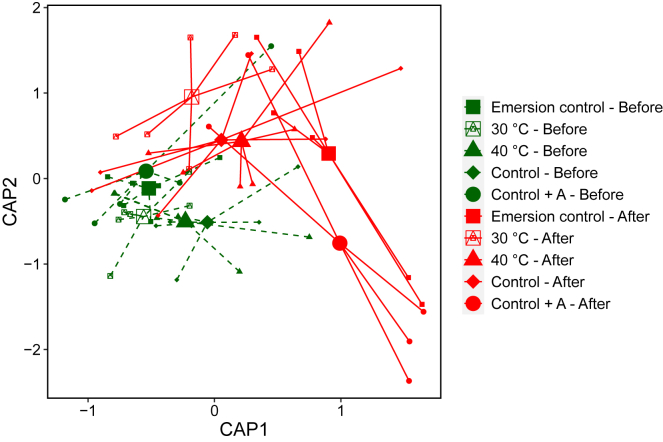
Ordination plot of bacterial community composition Canonical analysis of principal co-ordinates (CAP) plot calculated from Weighted-Unifrac distances of 16S rRNA bacterial data. Shapes indicate acclimation treatments and colors indicate before (green, dashed lines) and after (orange, solid lines) the 50°C heatwave. N = 6.

The 50°C heatwave caused a significant shift in the bacterial community composition, although there were no effects of heatwave acclimation (Figure 5; PERMANOVA F_3,47_ = 5.6, p < 0.01). These shifts were caused by significant changes in the relative abundance of 70 bacterial amplicon sequence variants (ASVs), 50 of which decreased in relative abundance (DESEQ p < 0.05). Spirochetes were the dominant group that declined in relative abundance following the heatwave, with a mean (±SE) decline in log fold decrease of −1.6 (±0.01). The greatest increases in relative abundance following the heatwave occurred in bacterial ASVs assigned to the Alteromonadaceae with a mean (±SE) log fold increase of 4.15 (±0.12) and Vibrionaceae families with a mean log fold increase of 3.5 (±0.12).

**Figure 6:**
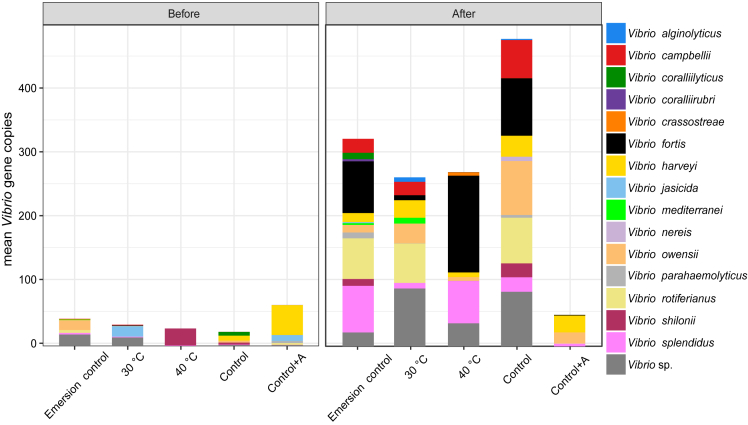
Vibrio bacterial community composition Bar plot showing the mean Vibrio gene copies as determined by qPCR per treatment, with colors indicating the relative abundance of Vibrio OTUs identified to the species level as determined by *hsp60* gene sequencing.

**Figure 7:**
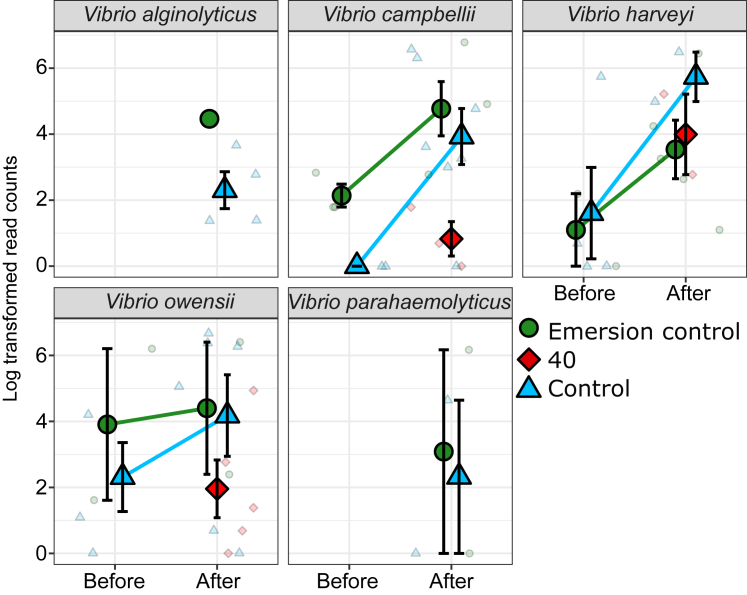
Abundance of members of the *Vibrio harveyi* clade Mean ± SE log reads from *hsp60* gene sequencing of the five members of the *Vibrio harveyi* clade before and after the 50°C heatwave. Colors and shapes indicate the acclimation treatments of oysters, small points indicate raw data points. In a few cases, before *hsp60* copy numbers were below detection limits.

The heatwave also significantly altered the Vibrio community composition within oysters as determined by *hsp60* gene sequencing (PERMANOVA F_1,35_ = 2.7, p < 0.01). There were no significant differences in Vibrio community composition among acclimation treatments prior to the heatwave (PERMANOVA p > 0.05). Following the heatwave, there was a significant increase in multiple Vibrio operational taxonomic units (OTUs), with the greatest increases occurring in the Control and Emersion control treatments (Figure 6) with 35 and 43 Vibrio OTUs increasing in relative abundance, respectively. This is in contrast to the 40°C acclimation treatment, in which only three Vibrio OTUs significantly increased in relative abundance following the heatwave. The largest significant (DESEQ P_adj_ < 0.05) increases in abundance in the Control treatment were Vibrio OTUs assigned as *Vibrio rotiferianus*, *Vibrio splendidus*, and *Vibrio fortis.* The largest increases in abundance in the Emersion control treatment were Vibrio OTUs assigned as *V. splendidus*, *V. fortis*, and *Vibrio parahaemolyticus.* There were also significant increases in five bacteria from the *Vibrio harveyi* clade including *V. harveyi, Vibrio owensii, Vibrio campbellii, Vibrio alginolyticus*, *and V. parahaemolyticus* in the Emersion control and in the Control treatment following the heatwave (Figure 7). No OTUs from the five *V. harveyi* clade species were detected in oysters from the 40°C acclimation treatment before the heatwave, and only three of the five were detected following the heatwave (*V. harveyi, V. owensii,* and *V. campbellii*). There were significantly less reads (K-W test; *Χ*^2^ = 4.3, p < 0.05) of bacteria from the *V. harveyi* clade detected after the heatwave in oysters from the 40°C acclimation treatment compared to the Emersion control and Control treatments (Figure 7).

## Discussion

Studies focusing on heatwave acclimation have largely avoided disease and its potential role in heatwave survival. We show that prior acclimation to elevated temperatures can reduce heatwave-induced mortality in intertidal invertebrates by improving their capacity to avoid proliferating pathogenic bacteria. Following a 50°C heatwave, oysters acclimated at 40°C had greater survival compared to non-acclimated oysters. Notably, oysters that had experienced no acclimation but were treated with antibiotics (Control + antibiotics) also experienced significantly less post-heatwave mortality, indicating a microbiological contribution to mortality. Acclimated oysters had a stronger immune response facilitated by more granulocytes; this likely contributed to acclimated oysters avoiding increases in potentially pathogenic bacteria such as those from the *V. harveyi* and *V. splendidus* clades. Non-acclimated oysters (Control and Emersion control) experienced the greatest increases in OTUs from the *V. harveyi* clade. Bacteria from this clade have previously been implicated in oyster mortality^19^^,^^33^ and are well established pathogens in aquaculture.^23^^,^^27^ Our findings suggest oysters that were not acclimated did not have the capacity to repel these increases in bacteria and subsequently may explain the greater mortality. Our findings highlight the previously overlooked role of bacterial infection in heatwave deaths and acclimation in intertidal habitats.

### Oyster immune responses to acclimation

Our study demonstrated the capacity for oysters to acclimate to thermal stress. Within 4 weeks, *S. glomerata* were able to acclimate and significantly reduce heatwave mortality. We found that acclimated oysters had more hemocytes in their hemolymph, including more granulocytes, compared to the Control and Emersion control treatments following the heatwave. There was no increase in the number of circulating hemocytes in oysters in both the Control and Emersion control treatments following the heatwave. Previous work on non-acclimated oysters has shown that more hemocytes are not always produced following a heatwave, and may in-fact decrease as cells die from heat stress.^34^^,^^35^^,^^36^ We observed a trend of decreasing numbers of granulocytes in Control oysters following the heatwave, perhaps indicating granulocyte death, or migration to sites of bacterial infection.

Responses in the oyster immune system are likely to be underpinned by cellular and physiological processes. Heat acclimation can occur by preventing the degradation of DNA and cell death by producing HSPs.^13^^,^^37^ Acclimated oysters may have these protective mechanisms, especially at 40°C allowing the production of granulocytes. While acclimation and the production of HSPs is generally considered to be energetically costly,^38^ we found no observable compromise with growth in the acclimation treatments; however, we did find that growth was more consistent in the Control treatment. It is possible that a longer time period would be needed to overcome the variability in growth measurements. Our results still indicate that heatwave acclimation may not come at a large compromise to growth.

Antibiotic addition led to the greatest granulocyte counts. This may be because oysters were triggered to produce hemocytes because of the heatwave, yet they were not required to phagocytize any bacteria and therefore were remaining in the blood and accumulated. Antibiotics are also known to trigger an immune response in mice models by altering the metabolome (Sun 2019); a similar process may be occurring in this study. Our 16S and *hsp60* gene sequencing and qPCR data show that the antibiotic treatments were highly successful at reducing the abundance of some bacteria. Survival of the Control oysters treated with antibiotics indicates that bacterial pathogens were likely responsible for oyster death following the heatwave. These findings are supported by previous work that has found Pacific oysters *Crassostrea gigas* had significantly greater rates of survival following a marine heatwave when treated with antibiotics.^19^ Our study goes a step further, however, and shows that equal levels of survival can be achieved by acclimation.

### Bacterial communities and potential pathogens following acclimation and heatwaves

Bacterial community composition was significantly altered by the 50°C heatwave, characterized by a jump in bacterial diversity. There was an increase in the Vibrionaceae and Alteromonadaceae families. Vibrio are well known marine pathogens,^39^ while the Alteromonadaceae have been described as pioneering bacteria,^40^ suggesting they are claiming space and nutrients left by the spirochetes that declined in response to the heatwave. Vibrio analysis with *hsp60* sequencing showed a significant increase in Vibrio OTUs in the Control and Emersion control treatments, but far smaller increases in these bacteria in oysters from the 40°C acclimation treatment. The Vibrio ASVs increasing in the Control and Emersion control oysters included significant increases in Vibrio bacteria from the Harveyi clade including *V. harveyi, V. owensii, V. campbellii, V. alginolyticus*, *and V. parahaemolyticus*, in addition to *V. splendidus* which are all known pathogens of oysters in aquaculture.^23^^,^^27^^,^^41^

Vibrio bacteria from the *V. harveyi* clade are known pathogens of marine animals and humans. *Vibrio haryevi* and the closely related *V. campbellii* bacteria produce serine proteases, metalloproteases, and cysteine proteases that are largely responsible for their virulence.^42^ The pathogenicity of Vibrio toward oysters has also shown to be enhanced at higher temperatures.^43^ The increased presence of these putative pathogens in oysters from the Control and Emersion control treatments suggests they may be responsible for the increased mortality after the heatwave.

Our results provide correlative evidence that oyster mortality occurred when Vibrio bacteria increased in relative (*16S, hsp60*) and absolute (qPCR) abundance. This increase appears to coincide with a decrease in spirochetes, a common oyster symbiont or mutualist.^39^^,^^44^^,^^45^ Recent research into the Pacific oyster mortality syndrome (POMS) revealed that bacterial infections by Vibrio resulted in oyster mortality following immune suppression by the OsHV-1 virus.^24^ Increasing Vibrio abundance may have come at the expense of other, potentially beneficial, or at least benign bacteria. This likely translated to mortality in oysters that had received no acclimation (Control and Emersion control) because they were unable to mount an immune defense against the increase in pathogens as evidenced by their unchanged and low levels of granulocytes, compared to oysters that were acclimated, and increased their granulocytes following the heatwave. Bacterial communities in oysters were not different among treatments prior to the heatwave, indicating that acclimation prior to the heatwave had little effect on bacterial communities. Rather, survival was determined by how oysters responded to the increasing bacteria following the heatwave. The oyster aquaculture industry is globally vulnerable to disease outbreaks, often following summer heatwaves.^25^ Our findings can lead to the practical development of strategies to reduce oyster disease following heatwaves, and can likely be applied across the industry to other aquaculture species vulnerable to Vibrio infection.

Previous work has focused on the ability for marine organisms to acclimate to heat stress by preventing the degradation of DNA and other protein damage by producing HSPs.^13^^,^^37^ This focus has largely overlooked the role of bacterial infection and disease in heatwave mortality. Our study has shown that a strong immune response may have been primed by acclimation, and this allowed the acclimated oysters to prevent the growth of putative pathogens following the atmospheric heatwave. Previous work has highlighted the capacity of marine invertebrates to respond to heatwaves with increased immune responses. For example, the pearl oyster *Pinctada maxima* genes encoding for the multifunctional protein with antimicrobial activities *Ubiquitin*^46^ were significantly upregulated in response to marine heatwaves.

### Conclusions

Our study shows that thermal acclimation reduced the presence of potential pathogens following the heatwave. This was likely due to acclimation increasing the ability of oysters to defend against potential pathogens. While previous work has shown that acclimation can reduce DNA damage, it is likely that maintaining the functions of the immune system following a heatwave is the difference between death and survival. These findings support the growing body of evidence that when considering how climate change will affect marine organisms, interactions between the host and microbiome must be considered, rather than investigating host responses alone. In conclusion, we have shown that increased survivorship following an acute heatwave may be due to an increased ability to repel the potentially pathogenic bacteria. This study partly overturns the paradigm that thermal acclimation is solely achieved by preventing cellular damage, and instead, shows that thermal acclimation may also enhance capacity to limit increases and susceptibility to the pathogenic microorganisms that cause mortality.

## STAR★Methods

### Key resources table

**Table:** 

REAGENT or RESOURCE	SOURCE	IDENTIFIER
**Deposited data**		

16S sequence data	NCBI	**PRJNA945181**
Hsp60 sequence data	NCBI	**PRJNA945181**

### Resource availability

#### Lead contact

Further information and requests for resources and reagents should be directed to and will be fulfilled by the lead contact, Elliot Scanes (elliot.scanes@uts.edu.au).

#### Materials availability

This study did not generate new unique reagents.

### Experimental model and subject details

One-thousand individuals of adult *S. glomerata* were sourced from Camden Haven Oyster Supply (31°38'28.8"S, 152°49'51.8"E) at an age of approximately six months, with an initial mean shell area of 490.07 ± 8.33 mm^2^ (Mean ± SE). Oysters were selected at this size because they have been shown to be vulnerable to atmospheric heatwaves.^31^ The oysters were transported to the Sydney Institute of Marine Science (SIMS; 33°50'20.3"S 151°15'17.9"E), Mosman NSW during the Austral autumn of 2022 and allowed to acclimate to laboratory conditions for one week. Following one week’s acclimation, three replicates of 250 oysters were consecutively assigned into five groups and placed into labelled mesh bags (15 groups total), and then placed into three continuous flow-through 55 L tanks with a flow rate of 2 L minute^-1^. This resulted in three tanks each containing 250 oysters, assigned into the 5 acclimation treatments (50 oysters each). The tanks were supplied with 100 μm filtered seawater drawn from the adjacent Sydney Harbour estuary at an average (± SE) temperature of 22.5 ± 0.1°C, salinity of 33.33 ± 0.08 PSU, and pH of 8.13 ± 0.01. Ten individuals from each group were tagged using glue-on shellfish tags (Hallprint Co.).

These groups represented the acclimation treatments called: Control, Control + antibiotic (Control +A), Emersion control, 30°C acclimation, and 40°C acclimation.

### Method details

#### Acclimation treatments and growth

Oysters were subjected to four weekly emersion exposures at the three treatment levels: Emersion control (ambient temperature; 23°C), 30°C, 40°C. These three treatments occurred while the oysters were emersed (out of the water) with oysters in the Control and Control + antibiotic treatments remaining submerged. Temperatures were selected to cover the range experienced on Australian shorelines.^12^ The first acclimation consisted of treatments being incubated for 1.5 hours in laboratory incubators (Ratek OM15). The length of the treatments was increased to 2 hours for the following three acclimations. The time was increased to reduce the shock on oysters in the first aclimation. These heat exposures will be referred to as “acclimation treatments”.

Prior to each exposure session, all oysters were removed from their tanks to record their growth and check for mortality. For growth measurements, the ten tagged oysters were photographed weekly, whereby the upper valve of the oysters was kept upright, perpendicular to the camera, with images of the shell area analysed using ImageJ (v1.53k).^47^ Dead oysters were counted and discarded, as were any individuals with open shells. Some shell edges were damaged during this process affecting growth measurements at the week 3 timepoint. Growth was calculated as daily rate of change in each oyster’s shell area from the previous measurement point.

#### Simulated atmospheric heatwave and survival

A simulated heatwave was performed in the fifth week of the experiment (one week after the final acclimation treatment), where all treatments were incubated at 50°C for 2 hours in three laboratory incubators, one for each tank. Temperatures of 50°C have previously been measured during atmospheric heatwaves in the habitat of *S. glomerata*^12^ and have caused mortality in *S. glomerata* in previous studies.^31^ Following exposure to the heatwave, the bags from each treatment containing 50 oysters were returned into fifteen 10 L enclosed tanks, which were aerated with air stones. Each replicate treatment was contained within its own tank to reduce risk of cross-contamination. An antibiotic mixture of Penicillin-Streptomycin was added to the Control + antibiotic tanks to give a concentration of 33 Penicillin units and 0.03 mg of Streptomycin per mL of tank water. Oysters remained in the 10 L tanks for 48 hours during their recovery from the heatwave. Water was completely changed in each tank 24 hours after the heatwave, with antibiotics re-added to the Control + antibiotics at the same concentrations. Survival was recorded following two days, after which the oysters were returned to their original flow-through tanks and then checked again after 8 and 16 days.

#### Sample analysis

##### Tissue and haemolymph extraction

Both before and two days after the simulated heatwave, gill tissue and haemolymph samples were taken from 2 shucked oysters per replicate treatment. Oysters were shucked with a sterilised shucking knife, and using a syringe, 100μL of haemolymph was drawn from the pericardial sack and placed on ice inside a centrifuge tube. Haemolymph samples were then vortexed with 8μL of 25% filtered glutaraldehyde (2% final concentration) and snap-frozen in liquid nitrogen (LN_2_) before being placed in a -80°C freezer. Gill tissue samples were extracted using sterile scissors and placed into a 1.8 mL CryoPure tube, for subsequent DNA extraction, and then snap-frozen in LN_2_ before being placed in a -80°C freezer.

##### Haemocyte counts

Hemocyte phagocytosis is the major immune response in oysters,^25^ with granulocytes identified as the cells responsible for phagocytosis. Therefore, to measure the capacity of the oyster immune system, haemocytes were counted using flow-cytometry. Haemolymph samples were thawed, dyed with SYBR green and then analysed using a flow-cytometer (Cytoflex LX) on a 525/40 channel following the previously described STAR Methods for *S. glomerata*.^48^ Total haemocytes were separated from background noise by gating cells. This was confirmed against un-stained samples, which showed similar amounts of noise in this region. Small cells were separated from large cells based on SSC and FSC over height (Figure S2). These gates and patterns of cell distribution were consistent with those previously reported by.^48^

##### DNA extraction, 16S rRNA and hsp60 sequencing

DNA from oyster gills was extracted using the Qiagen DNeasy Blood and Tissue Kit (Qiagen Australia, Chadstone, VIC), according to the manufacturer’s instructions. Control DNA kit extractions were extracted and sequenced together with our samples. To characterise bacterial community composition, the V3–V4 region of the bacterial 16S rRNA gene was amplified using the Bakt_341F and Bakt_805R primer set (Herlemann et al., 2011), with the following cycling conditions: 95°C for 3 minutes followed by 25 cycles of: 95°C for 30 seconds, 55°C for 30 seconds, 72°C for 30 seconds, and then 72°C for 5 minutes with a final hold at 4°C (Illumina Co., 2013). To allow for deeper examination of the oyster-associated *Vibrio* community with improved taxonomic resolution, we also used a custom *Vibrio*-centric *hsp60* amplicon sequencing assay using the Vib-hspF3-23 and Vib-hspR401-422 primer pair with the same PCR cycling condition as described previously.^49^

Amplicon sequencing of both 16S rRNA and HSP60 was performed using the Illumina Miseq platform (2x300bp) following the manufacturer’s guidelines (Ramaciotti Centre for Genomics, University of New South Wales, Sydney, NSW, Australia). Raw data files in FASTQ format were deposited in NCBI Sequence Read Archive (SRA) under Bioproject number NCBI: **PRJNA945181**.

Raw demultiplexed 16S rRNA data was processed using the Quantitative Insights into Microbial Ecology (QIIME 2 version 2019.1.0) pipeline. Briefly, paired-end sequences were imported, trimmed and denoised using DADA2 (version 2019.1.0).^50^ Sequences were identified at the single nucleotide level (Amplicon Sequence Variants; ASV) and taxonomy was assigned using the classify-sklearn qiime feature classifier against the Silva v138 database.^51^ Rarefaction plots were used to check sequencing depth, and data were rarefied to 3900 sequences per sample, which excluded two samples and the two kit controls.

Raw demultiplexed *hsp60* data was processed as previously described.^49^ Reads were joined using Flash^52^ and the resulting fragments were trimmed with Mothur.^53^ Fragments were clustered into operational taxonomic units (OTUs) at the 97 % threshold and chimeric sequences were removed using vsearch.^54^ Taxonomy was assigned to fragments using QIIME^55^ and the RDP classifier^56^ according to a custom *Vibrio*-*hsp60* reference dataset.^49^ OTUs with less than 25 total reads were removed and sequences were normalized to the number of sequences per sample to produce relative abundance.

##### Quantitative PCR (qPCR)

Abundances of *Vibrio* bacteria were quantified using the primer pair Vib1-f and Vib2-r,^57^^,^^58^ which amplify the 16S rRNA genes specific to the *Vibrio* genus. qPCR assays were prepared with an epMotion 5075I Automated Liquid Handling System and performed on a Bio-Rad CFX384 Touch Real-Time PCR Detection System, with three technical replicates, a standard curve, and negative controls. A melting curve was also added to the end of the *Vibrio* specific assay to confirm the presence of a single PCR product. The reaction mixture for each assay included: 2.5 μL iTaq Universal probes SMX or iTaq Universal SYBR Green SMX (Bio-Rad), 0.2 μL of each 10 μM forward and 10 μM reverse primer, 1 μL of template DNA, and 1 or 1.1 μL of sterile water for a final reaction volume of 5 μL.

The *Vibrio* specific 16S rRNA qPCR was performed using the following cycling conditions: 95°C for 3 minutes followed by 40 cycles of 95°C for 15 seconds and 60°C for 1 minute, followed by a melting curve to confirm the amplification of only a single product. For quality control, the coefficient of variation (CV) was calculated for the qPCR technical triplicates, and where necessary, triplicates with a CV of greater than 2 % had a technical replicate removed from the analysis.

### Quantification and statistical analysis

Statistical analyses were performed using R software (v3.4.0; R Core Team, 2021) with alpha set at 0.05 for statistical significance. The survival of oysters throughout the experiment, including after the heatwave, was analysed using Cox’s proportional hazard regression model where hazard is an indicator of mortality risk due to the contribution of a factor.^59^ Tank was confirmed to not be a significant factor contributing to oyster mortality, then the Cox’s proportional hazard model was used to determine the contribution of Acclimation Treatments (5 levels) to oyster death.

Growth data was analysed by determining the amount of growth for each oyster between the previous weekly measurement point, and the time at which measurements were taken. This value was then standardised by the number of days to give shell area growth in mm^2-d^. ANOVA was then used to compare the rate of growth for oysters with “Acclimation treatment” and “Time” as the fixed factors and “Tank” as a random nested factor in “Treatment”.

Data from flow cytometry and Alpha diversity metrics from 16S sequencing were confirmed to meet the assumptions of ANOVA and was subsequently analysed using a two-way ANOVA with “Acclimation treatment”, “Heatwave (before or after)” as fixed factors. Data was confirmed as normal by the visual observations of frequency histograms and the Shapiro-Wilk normality test. Residual plots were used to check model fit. Subsequent Tukey’s HSD *post hoc* test were used to investigate the pairwise differences between treatments.

*Vibrio* abundance from qPCR data showed a Poisson distribution and were therefore analysed using a negative binomial GLM with “Acclimation treatment” and “Time” as the fixed factors. The ‘ANOVA’ function (CAR package) was then used to conduct Wald (type II, *χ*^2^) tests of analysis of deviance on linear models to determine *p* values. Parson's residual plots were used to check the model fit and the residual deviance and residual degrees of freedom were used to check for over-dispersion (Binominal GLMs) (Resid.Dev/Resid.Df for all analyses was < 1). For all GLM analyses the “Tank” factor was found to not contribute to the model (P>0.1) so was removed. All linear regression analyses were done using the lmer package in R software version 3.4.0 (R Development Core Team 2015). Tukey’s HSD *post hoc* test were used to investigate the pairwise differences between treatments.

For *16S* rRNA and *hsp60* gene sequencing data, PERMANOVA was used to determine significant differences between treatment levels using a two-way design with “Acclimation treatment”, “Heatwave (before or after)” as fixed factors; these analyses were then repeated with the Control + antibiotics treatment removed. DESeq analysis was used to determine significant effects of the heatwave on the relative abundance of ASVs and OTUs, DESeq comparisons were made before an after the heatwave for all individual treatments. A Kruskal-Wallis test was used to determine significant differences among the *hsp60* log transformed reads of Harveyi clade *Vibrios* after the heatwave for the Emersion control, Control and the 40°C treatments. Only the “after” time point could be used because no OTUs from the *Vibrio Harveyi* clade were detected before the heatwave in the 40°C treatment. For all statistical analyses, details of results are provided in the results section, the number of replicate oysters (n) can be found within figure legends for each analysis.

## Data Availability

• All 16S and hsp60 gene sequences have been deposited at the NCBI data repository and are publicly available as of the date of publication. Accession numbers are listed in the key resources table. All remaining data can be shared by the lead contact following request. • This study did not generate any original code. • Any additional information required to reanalyze the data reported in this paper is available from the lead contact upon request.
