# Supplementary material for: Acclimation in intertidal animals reduces potential pathogen load and increases survival following a heatwave
Source: iScience. 2023 May 4;26(6):106813. doi: 10.1016/j.isci.2023.106813 (PMC10199257; doi:10.1016/j.isci.2023.106813)
Supplement: Document S1. Figures S1–S3 [file mmc1.pdf]

iScience, Volume *26*

Supplemental information

Acclimation in intertidal animals reduces potential pathogen load and increases survival following a heatwave

Elliot Scanes, Nachshon Siboni, Brendon Rees, and Justin R. Seymour

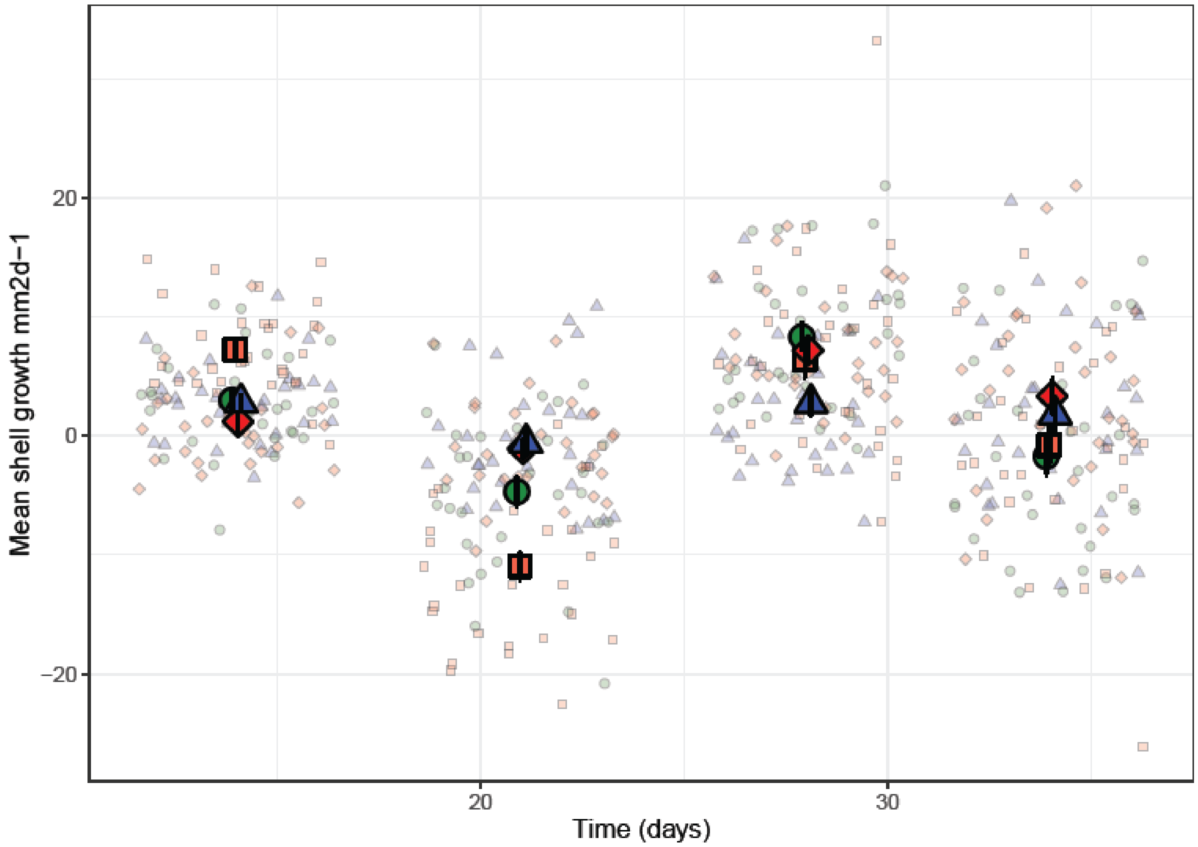

Figure S1. Oyster shell growth displayed as the difference in shell size (mm^2) between measurement points. Data was then standardised to growth per day. Large points indicate Mean ±SE, smaller points indicate raw data points. Relates to Results section “Oyster growth”.

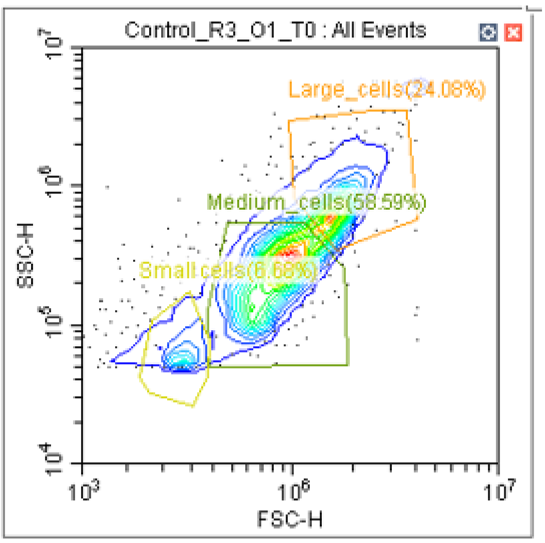

Figure S2. Density plot of all events from the haemolymph of control oysters. The "Large cells" gate was used to determine granulocyte counts. Relates to STAR methods section "*Haemocyte counts*" and Figure 2.

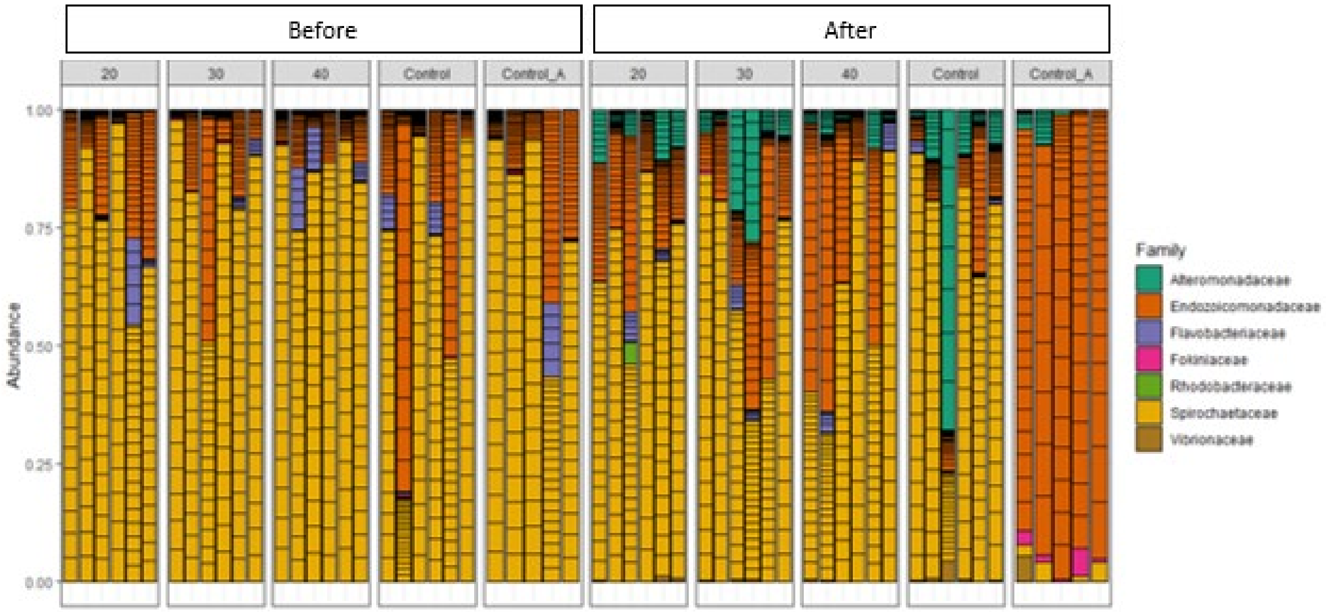

Figure S3. Barplot of the relative abundance of bacterial ASVs in each sample to a taxonomic resolution of Family. Data have been filtered to exclude families with >2% relative abundance. Samples are divided by their acclimation treatments and before or after the heatwave. Relates to Results section "*Bacterial community composition*".
